# Massively parallel sequencing of single cells by epicPCR links functional genes with phylogenetic markers

**DOI:** 10.1038/ismej.2015.124

**Published:** 2015-09-22

**Authors:** Sarah J Spencer, Manu V Tamminen, Sarah P Preheim, Mira T Guo, Adrian W Briggs, Ilana L Brito, David A Weitz, Leena K Pitkänen, Francois Vigneault, Marko PJuhani Virta, Eric J Alm

**Affiliations:** 1 Computational and Systems Biology, Massachusetts Institute of Technology, Cambridge, MA, USA; 2 Department of Biological Engineering, Massachusetts Institute of Technology, Cambridge, MA, USA; 3 Department of Food and Environmental Sciences, University of Helsinki, Helsinki, Finland; 4 School of Engineering and Applied Sciences, Harvard University, Cambridge, MA, USA; 5 Department of Physics, Harvard University, Cambridge, MA, USA; 6 AbVitro Inc., Boston, MA, USA; 7 Department of Civil and Environmental Engineering, Massachusetts Institute of Technology, Cambridge, MA, USA; 8 The Center for Microbiome Informatics and Therapeutics, Massachusetts Institute of Technology, Cambridge, MA, USA; 9 The Broad Institute of MIT and Harvard, Cambridge, MA, USA

## Abstract

Many microbial communities are characterized by high genetic diversity. 16S ribosomal RNA sequencing can determine community members, and metagenomics can determine the functional diversity, but resolving the functional role of individual cells in high throughput remains an unsolved challenge. Here, we describe epicPCR (Emulsion, Paired Isolation and Concatenation PCR), a new technique that links functional genes and phylogenetic markers in uncultured single cells, providing a throughput of hundreds of thousands of cells with costs comparable to one genomic library preparation. We demonstrate the utility of our technique in a natural environment by profiling a sulfate-reducing community in a freshwater lake, revealing both known sulfate reducers and discovering new putative sulfate reducers. Our method is adaptable to any conserved genetic trait and translates genetic associations from diverse microbial samples into a sequencing library that answers targeted ecological questions. Potential applications include identifying functional community members, tracing horizontal gene transfer networks and mapping ecological interactions between microbial cells.

## Introduction

‘Who is doing what' is a major open question in microbial ecology. While 16S ribosomal RNA (rRNA) sequencing can answer the ‘who', and shotgun metagenomics can partially address the ‘what', connecting the two is difficult. In recent years, investigators have tried different approaches to ask targeted ecological questions at the resolution of single cells. The most common approach to connect phylogeny with function combines single-cell FACS sorting with whole-genome amplification and PCR screening for target genes (Stepanauskas and Sieracki, 2007; Siegl and Hentschel, 2010; Martinez-Garcia *et al.*, 2012; Bayer *et al.*, 2013). Other methods isolate single cells using microfluidics, then screen for target genes either in microfluidic chambers or on primer-coated beads (Ottesen *et al.*, 2006; Zeng *et al.*, 2010; Tadmor *et al.*, 2011). There are also variants of fluorescence *in situ* hybridization that show colocalization of target gene probes (Gieseke *et al.*, 2003; Valm *et al.*, 2011; Baptista *et al.*, 2014). Despite these advances, current methods face persistent limitations in throughput, reagent costs and labor requirements. Motivated by this technology gap, we developed a cost-effective and highly parallel technology to answer ‘who is doing what' in high throughput in any microbial community.

Here, we present epicPCR (Emulsion, Paired Isolation and Concatenation PCR), a novel method for recovering linked phylogenetic and functional information from millions of cells in a single experiment. Emulsion-based techniques provide a simple way to partition bulk reactions into millions of individual reactions, each within a single droplet. This approach is not new, and has been used by sequencing platforms such as 454 and Ion Torrent to prepare templates for sequencing. Emulsion techniques have also been used in studies of human haplotypes from single cells and studies of single-cell immunology (Turner and Hurles, 2009; Turchaninova *et al.*, 2013) using emulsions in combination with fusion PCR, a technique originally developed for preparing fusion proteins (Yon and Fried, 1989).

A significant challenge in translating emulsion technology to microbiology is the difficulty of microbial cell lysis. The epicPCR methodology we present here permits efficient cell lysis by isolating cells in emulsion droplets before PCR and encapsulating them in a hydrogel matrix (Tamminen and Virta, 2015). This matrix is dense enough to hold bacterial genomes in place after hydrogel bead recovery, but loose enough to allow enzymes and primers to diffuse through (Holmes and Stellwagen, 1991; Umbarger *et al.*, 2011). Hydrogel beads are then loaded into a second emulsion where amplified target genes become physically linked by fusion PCR.

We demonstrate epicPCR by detecting a rare sulfate-reducing cell population among the microbial diversity of a freshwater lake, sequencing 16S rRNA genes from cells containing the dissimilatory sulfate reductase gene *dsrB* (Leloup *et al.*, 2004). We confirm that the observed phylogenetic distribution of *dsrB* genes matches predictions based on observed geochemistry, while also revealing previously undetected putative sulfate reducers. The efficiency of microbial cell lysis can be measured by comparing untargeted epicPCR with bulk 16S rRNA gene data. Our bulk emulsion design can query hundreds of thousands of cells in parallel with costs comparable to one genomic library prep, increasing throughput and reducing expense compared with existing methods. This adaptable method can translate genetic associations from any sample into a sequencing library that answers targeted ecological questions.

## Materials and methods

### Lake water sample collection and quantification

Lake water was collected from Upper Mystic Lake (~42′26.155″ N, 71′08. 961″ W) near Winchester, Massachussetts, USA on 12 August 2013. Duplicate samples were taken from depths of 2 and 21 m, with 15 ml of lake water immediately placed in 25% glycerol and frozen on dry ice for transport and subsequent storage at −80 °C. Approximate cell counts were determined using one of the duplicate samples for each depth. Samples were diluted, fixed with formalin and stained with DAPI (4',6-diamidino-2-phenylindole) to perform cell counts on a fluorescent microscope. Description of DNA extraction and bulk 16S rRNA gene library preparation for these samples can be found in Supplementary Methods.

### Polymerization and lysis of lake water samples

We thawed a glycerol stock of lake water and suspended 14 million cells in nuclease-free water. This suspension was combined with ammonium persulfate, acrylamide and *N*,*N*′-bis(acryloyl)cystamine as a crosslinker. The 255 μl aqueous mixture was applied to 600 μl Span 80/Tween-80/Triton X-100 emulsion oil (Williams *et al.*, 2006) and vortexed for 30 s, which produced ~500 million droplets (based on 10 μm average droplet diameter, see Supplementary Information). We added a small volume of tetramethylethylenediamine to catalyze the polymerization and vortexed for an additional 30 s, and then let the emulsion polymerize for 90 min. Polyacrylamide beads were extracted with diethyl ether, then resuspended in 1 ml 1 × TK buffer and filtered through a 35 μm cell strainer. Detailed methods for these steps are available in the Supplementary Information.

We performed epicPCR assays on the polyacrylamide beads both with and without additional lysis reagents. For the beads with additional lysis treatment, we added 0.8% Ready-Lyse Lysozyme (35 000 U μl^−1^; Epicentre, Madison, WI, USA) to polyacrylamide bead aliquots and incubated at 37 °C overnight. Each aliquot was centrifuged and resuspended in 1 × TK buffer, and then treated with 20% (v v^−1^) proteinase K (1 mg ml^−1^, Sigma, St Louis, MO, USA) and 0.8% (v v^−1^) Triton X-100. The samples were incubated at 37 °C for 30 min, and then at 95 °C for 10 min. Following treatment, polyacrylamide beads were again centrifuged and resuspended in 1 × TK buffer for the epicPCR library preparation.

### Preparation of synthetic control polyacrylamide beads

We amplified DNA segments with acrydited 5′ ends and attached them to polyacrylamide beads to serve as synthetic positive and negative controls. To prepare these beads, we created a bulk emulsion with ~500 million droplets by vortexing for a total of 60 s, and diluted our acrydited DNA to load 100 molecules per droplet on average. In our negative control preparation, we added 0.7 μm 16 S-V4neg PCR product. In our positive control preparation, we added 0.7 μm 16 S-V4pos PCR product plus 0.7 μm dsrB-synth primer (PCR product and primer sequences in Supplementary Table S1, primers adapted from Wagner *et al.*, 2005). To prepare the polyacrylamide beads, we combined our acrydited DNA segments with an aqueous reaction mixture, emulsified the aqueous phase and polymerized the emulsion droplets as described in Supplementary Methods. Five rounds of centrifugation (12 000 *g* for 1 min) and removal of the low-molecular-weight polyacrylamide beads, followed by filtration through a 35 μm cell strainer, ensured a more even size distribution for the synthetic controls.

### epicPCR library preparation

**Figure 1:**
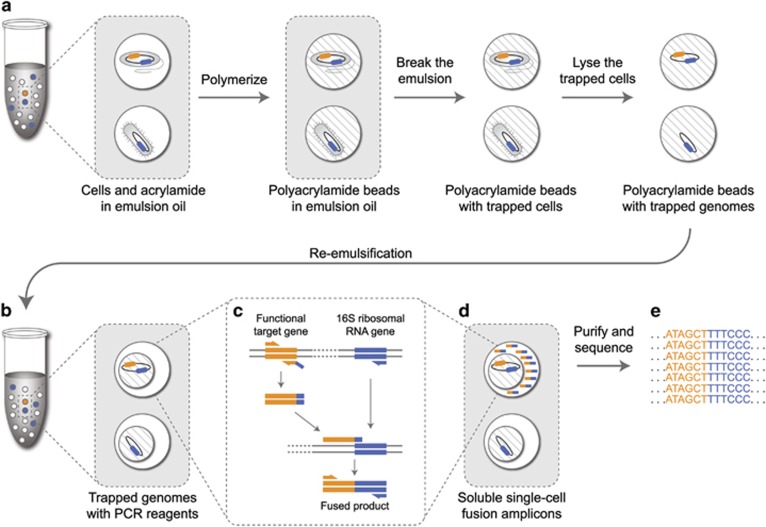
Workflow of epicPCR. (**a**) Microbial cells in acrylamide suspension are mixed into emulsion oil. The emulsion droplets are polymerized into polyacrylamide beads containing single cells. The emulsion is broken and the cells in the polyacrylamide beads are treated enzymatically to destroy cell walls, membranes and protein components, and expose the genomic DNA. (**b**) Polyacrylamide-trapped, permeabilized microbial cells are encapsulated into an emulsion with fusion PCR reagents. (**c**) Fusion PCR first amplifies a target gene with an overhang of 16S rRNA gene homology. With a limiting concentration of overhang primer, the target gene amplicon will anneal and extend into the 16S rRNA gene, forming a fusion product that continues to amplify from a reverse 16S rRNA gene primer. (**d**) The fused amplicons only form in the emulsion compartments where a given microbial cell has the target functional gene. (**e**) After breaking the emulsion, the fused amplicons are prepared for next-generation sequencing. The resulting DNA sequences are concatemers of the target functional gene and the 16S rRNA gene of the same cell.

First, we prepared an emulsion with polyacrylamide beads and fusion PCR primers to amplify the single-cell fusion templates. The PCR mix included 45 μl of polyacrylamide beads combined with PCR reagents and emulsion stabilizers (bovine serum albumin and Tween-20). We also added the three fusion primers (Figure 1b,Supplementary Figures S1A and D and Supplementary Table S2): 1 μm F1, 1 μm R2 and a limiting concentration of 10 nm R1-F2′ to bridge between the target gene and 16S rRNA genes. For these generic primer names refer to Supplementary Figure S1A; for specific experiments, please refer to Supplementary Figures S1C and D for primer names and Supplementary Table S2 for primer sequences. For PCRs with a soluble barcode-16S rRNA gene fusion (abbreviated barcode-16S), we added 100 fM fusionBarcode. Supplementary Table S3 presents an outline of primers used for different experiments and Supplementary Figures S1C and D show fusion construct designs. Supplementary Figure S2 shows the genomic context of the *dsrB* primers, adapted from Wagner *et al.* (1998) and Giloteaux *et al.* (2010)). The final aqueous PCR mix was added to 900 μl ABIL EM 90 emulsion oil (Williams *et al.*, 2006), vortexed and then aliquot into PCR tubes for thermocycling. Following amplification, aliquots were pooled, phase separated and purified with AMPure XP beads (see Supplementary Methods for detailed procedures and sample information).

Following this reaction, we added another set of primers to nest within the fused products and also block the amplification of unfused pieces (Supplementary Table S4). The nested PCR included standard PCR reagents combined with 0.3 μm forward and reverse nested primers (for specific experiments, please refer to Supplementary Figures S1C and D for primer names and Supplementary Table S4 for primer sequences) plus 3.2 μm each of U519F_block10 and U519R_block10, which are modified universal 16S rRNA gene primers (Lane, 1991) that prevent amplification of unfused pieces (Supplementary Figure S1B). The blocking primers were enhanced from the design presented in Wetmur *et al.* (2005) and Turchaninova *et al.* (2013) by the addition of 3′ 3-carbon spacers; these spacers show decreased degradation and increased blocking efficiency over 3′ phosphates (Cradic *et al.*, 2004). We combined the nested and blocking primers with purified fusion product from the previous reaction and ran quantitative PCRs to determine the number of amplification cycles to use for each sample. Using the quantitative PCR Ct values, we completed the final nested reaction, purified the products and amplified again with Illumina adapters (Supplementary Table S5). These adapters included a 3′ YRYR sequence to add template diversity to the amplicon library. Purified final libraries were sequenced on an Illumina MiSeq with 250 bp paired-end reads (see Supplementary Methods for detailed procedures).

### epicPCR sequence analysis and OTU clustering

The resulting sequence data were filtered for quality and expected fusion structure. Throughout the analysis, we frequently used functions from the software package QIIME; functions had default parameters unless otherwise specified (Caporaso *et al.*, 2010). After splitting samples by sample barcode, we stitched together forward and reverse reads and then filtered for quality (at Phred>Q20). Chimera checking was critical for our fusion constructs, thus we ran the non-reference-based identify_chimeric_seqs.py (-m usearch61). The remaining reads were trimmed to 121 bp of the 16S rRNA gene V4 region based on a conserved 16S rRNA gene V4 site (Baker *et al.*, 2003), and we discarded any reads that did not match our expected fusion bridge structure using custom python scripts (version 2.7; https://github.com/sjspence/epicPCR). To identify positive and negative control 16S rRNA gene sequences, we performed a targeted BLAST search against our synthetic 16S rRNA gene sequences.

For operational taxonomic unit (OTU) determination, we first collapsed identical droplet barcode-16S pairs into a single representative sequence using a custom python script (version 2.7; https://github.com/sjspence/epicPCR/blob/master/compressBar.py). This function controlled for droplets that amplified exponentially more than others because of the heterogeneous droplet volume. We then ran a series of QIIME functions that grouped 16S rRNA gene sequences into 97%, 95% and 80% identity clusters, picked representative sequences and assigned taxonomy based on the Greengenes and SILVA databases (DeSantis *et al.*, 2006; Caporaso *et al.*, 2010; Quast *et al.*, 2013). To facilitate visual comparison between samples despite different sequencing depths, we rarefied to the sample with the fewest reads; when we did not compare between samples, we presented the full read set (see Supplementary Methods for detailed procedures).

For tree construction, the 16S rRNA gene sequences picked by epicPCR were combined with bulk 16S rRNA gene sequence data of the sample. The epicPCR 16S rRNA gene sequences and bulk 16S rRNA gene sequences had been separately grouped into 95% and 80% identity clusters, and sequences from the respective clustering distances were combined. The sequences were aligned using SINA (Pruesse *et al.*, 2012). The tree was constructed using FastTree 2.1.7 (Price *et al.*, 2010).

For functional classification, the *dsrB* sequences were grouped into 95% identity clusters by uclust 1.2.22 and aligned to a *dsrAB* database (Müller *et al.*, 2015) using the NAST output option of usearch v.8.0.1517 (Edgar, 2010). A reference tree was constructed from the *dsrAB* database using FastTree 2.1.7 (Price *et al.*, 2010). Range and specificity of epicPCR primers (Supplementary Figure S1D,Supplementary Table S2 and Supplementary Table S4) was tested in an *in silico* PCR against the *dsrAB* database into two steps using the EMBOSS 6.5.7 primersearch tool with 20% mismatch cutoff (Rice *et al.*, 2000): first, we extracted *in silico* amplicons from the *dsrAB* database using the sequence of primer dsrB-F1 and segment 5′-TGCCTSAAYATGTGYGGYG-3′ from primer dsrB-R1. Subsequently, we extracted a subset from these *in silico* amplicons using primer segments 5′-VAGVATSGCGATRTCGGA-3′ from i_dsrB-F3 and 5′-TGCCTSAAYATGTGYGGYG-3′ from dsrB-R1. Complete matches of epicPCR *dsrB* fragments to the *dsrAB* database were identified using the grep tool from OS X Yosemite. Matches of bulk *dsrB* fragments (see Supplementary Methods and Supplementary Figure S3A for details of bulk *dsrB* sequencing) to the *dsrAB* database were identified using BLAST 2.2.30 (Altschul *et al.*, 1990) using a similarity cutoff of 70%. The *in silico* PCR results, epicPCR *dsrB* matches and bulk *dsrB* matches in the *dsrAB* database were visualized in a *dsrAB* reference tree using iTOL (Letunic and Bork, 2011) (Supplementary Figure S3B).

### Data access

The raw sequencing data from this study were submitted to the NCBI Sequence Read Archive (http://www.ncbi.nlm.nih.gov/sra) under the accession number PRJNA264605. The computational steps we used to process the data are detailed in a text file along with custom scripts available at https://github.com/sjspence/epicPCR.

## Results

### Benchtop emulsions enable genome capture and targeted sequencing of single cells within complex communities

epicPCR combines established methods for cell isolation, encapsulation and paired amplification. An overview of the method is as follows: an initial aqueous sample-in-oil emulsion generates ~500 million droplets, each about one nanoliter in volume, that contain single cells. These cells are loaded and dispersed assuming Poisson statistics, so that on average less than one droplet in 100 contains a cell. Each of these droplets also contains acrylamide monomers that polymerize and encapsulate cells upon addition of a catalyst, forming polyacrylamide beads (Figure 1a). The polyacrylamide hydrogel provides support for bacterial chromosomes and plasmids, preventing their diffusion when the trapped cells are combined in bulk and redistributed for fusion PCR. The diameter of the polyacrylamide beads typically ranges from 5 to 30 μm with most beads having a diameter around 10 μm, determined by light microscopy as described previously (Tamminen and Virta, 2015), with representative images in Supplementary Figure S4A.

Fusion PCR is performed on the hydrogel-trapped genomes in a secondary emulsion (Figure 1b and Supplementary Figure S4B) to ensure that each epicPCR is compartmentalized (Figures 1c and d). The protocol has been described previously (Turner and Hurles, 2009) and proceeds as a single reaction with an initial linear amplification of the 16S rRNA gene and a limited-cycle exponential amplification of a separate target gene. The limited-cycle exponential amplification is carried out using a primer pair where one of the primers has an overhang that is complementary with a part of the 16S rRNA gene. After this overhang primer is depleted, the complementary part will form a fusion amplicon with the 16S rRNA gene, and exponential amplification of the fusion amplicon proceeds.

Illumina adapters are subsequently added to pooled fusion amplicons in a bulk nested PCR (Figure 1e and Supplementary Figure S1B). Without refined molecular control, partially fused products could continue the reaction in bulk and destroy single-cell specificity. Aptamer-based hot start polymerase prevents partially fused products from extending, preserving single-cell specificity in the bulk reaction. Then, a saturating concentration of blocking primers anneals to and removes any partially fused pieces from the bulk library amplification (Wetmur *et al.*, 2005; Turchaninova *et al.*, 2013) (Supplementary Figure S1B). Collectively, the steps of this protocol are designed to preserve the individually fused information from single cells while maintaining high throughput.

### Spiking an environmental sample with synthetic control beads demonstrates high specificity of epicPCR

One exciting application of this technology is to link phylotype to function in a complex community. Here, we processed lake water from oxic and anoxic depths, and then used epicPCR to target cells harboring the dissimilatory sulfite reductase gene *dsrB*. Sulfate reduction is a process where microbial cells in anoxic conditions use sulfate as the terminal electron acceptor of their metabolism. We recorded the geochemistry of water from an urban lake by measuring sulfate, nitrate and oxygen at 1-m intervals down to 22 m (see Supplementary Information for details). At a 21- m depth, both oxygen and nitrate are depleted, but sulfate is still available as an electron acceptor (Supplementary Figure S5).

Our single-cell experimental design consisted of epicPCR assays on 2 and 21 m lake water with positive and negative spike-in controls. We produced spike-in controls by synthesizing polyacrylamide beads that contained covalently attached DNA amplicons. Negative control beads carried a mock-16S rRNA gene, whereas positive control beads had both a mock-16S rRNA gene (with a sequence distinct from the negative control beads) and a mock-*dsrB* sequence.

To compare the full 16S rRNA gene diversity present to the *dsrB*-carrying subpopulation, we completed both nonspecific and *dsrB*-specific epicPCR assays. Our nonspecific assay fused together 16S rRNA gene sequences with a synthetic amplicon carrying a random DNA barcode. The barcode, based on 20 degenerate nucleotides, was added at a concentration of 10 pm, which loads on average three molecules per 10 μm diameter droplet. As cell-containing and control polyacrylamide beads are all likely to be in droplets containing barcodes, we expected this reaction to result in fusions to all environmental, positive and negative control 16S rRNA gene sequences. Our *dsrB*-specific assay fused *dsrB* gene fragments with 16S rRNA genes present in the same droplet. We expected to observe only 21 m, anoxic species and positive control 16S rRNA gene sequences in our *dsrB*-fusion products.

**Figure 2:**
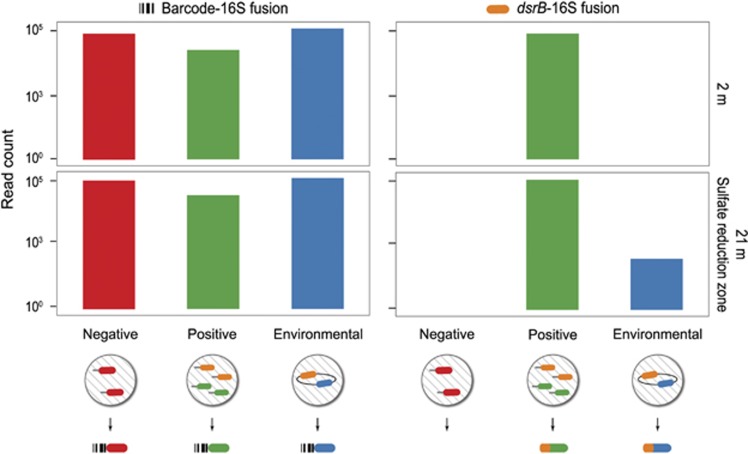
Specificity of epicPCR is tested in a series of experiments in which a random barcode or a *dsrB* gene fragment is fused with the 16S rRNA gene in an environmental sample that is spiked with negative and positive controls. Negative controls are synthetic polyacrylamide beads with attached mock-16S amplicons. In epicPCR, these beads result in a positive signal for barcode fusion but give no signal for *dsrB*-16S fusion. Positive controls are synthetic polyacrylamide beads with attached mock-16S and mock-*dsrB* amplicons. In epicPCR, these beads result in a positive signal for both barcode-16S and *dsrB*-16S fusions. For environmental cells from a freshwater lake, barcode-16S reactions capture the 16S rRNA gene diversity at both 2 and 21 m depths. Sulfate reduction takes place in the anoxic layers far below the surface, so *dsrB*-16S fusions only occur successfully at the 21 m depth.

Fusions to 16S rRNA genes from environmental cells matched our expectation that sulfate-reduction machinery would only occur at anoxic depths. We recovered *dsrB*-16S fusion amplicons from the 21- m depth, but detected no *dsrB*-16S rRNA gene fusions (abbreviated *dsrB*-16S) at 2 m (Figure 2). The depth specificity is not because of assay bias because 1 167 006 nonspecific barcode-16S fusions evenly captured both 2 and 21 m diversity.

As expected in our controls, we observed ubiquitous 16S rRNA gene fusions to the nonspecific barcode amplicon, but highly specific positive control amplification in *dsrB*-16S fusion products. Barcode-16S fusion products captured 388 768 reads containing the negative control 16S rRNA gene sequence and 70 154 reads containing the positive control 16S rRNA gene sequence. In contrast, the targeted *dsrB*-16S fusion design captured exclusively positive control 16S rRNA gene sequences—a total of 372 223 reads—with zero observations of the negative control 16S rRNA gene sequence, confirming the high specificity of the technique.

### Abundant phyla are consistently targeted by epicPCR

**Figure 3:**
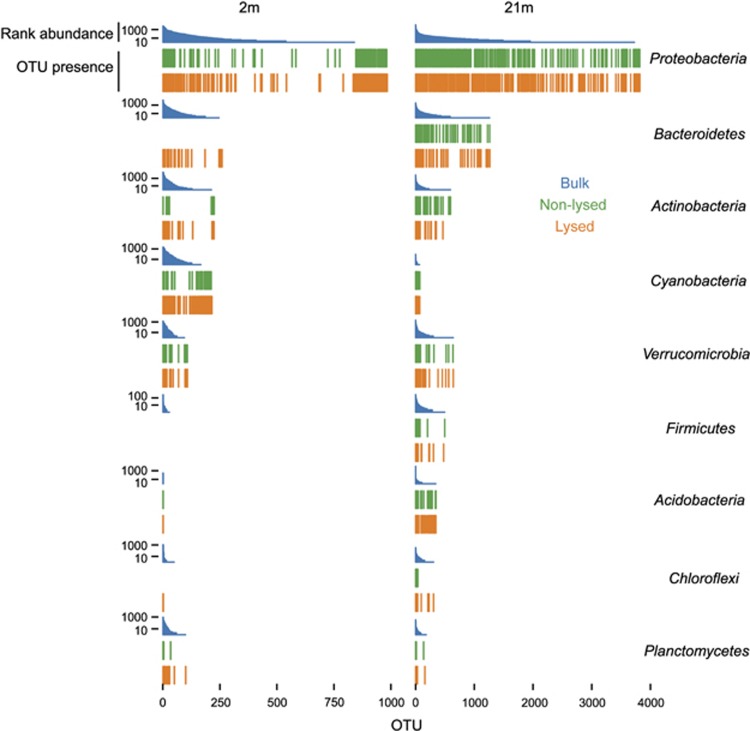
Bacterial groups recovered by a bulk 16S rRNA gene survey and epicPCR from the 2 and 21 m depths. OTU rank abundance of the bulk 16S rRNA sequencing is presented as blue histograms. Corresponding OTUs identified by epicPCR are presented as bars below the rank abundance histograms. This includes reactions with (yellow) and without (green) additional lysis reagents. epicPCR captures most phyla within a sample, regardless of cell structure or phylogeny. The use of additional lysis reagents, including lysozyme, proteinase K and detergents, increases the phylogenetic coverage of the assay for certain bacterial groups such as *Actinobacteria*, *Bacteroidetes*, *Chloroflexi*, *Cyanobacteria* and *Planctomycetes*.

Comparisons of the 16S rRNA gene diversity from barcode fusion and bulk 16S rRNA gene sequencing shows that epicPCR recovers all major phylogenetic groups, indicating that cells from most of these groups became successfully permeabilized in a replicated experimental setup despite variable cell wall structures (Figure 3 and Supplementary Figure S6). Treatment with lysozyme, proteinase K, detergents and heat permeabilized certain additional phyla relative to the standard epicPCR protocol.

Most dominant phyla were successfully permeabilized even without enzymatic treatment (Figure 3). However, certain phyla such as *Actinobacteria*, *Bacteroidetes, Cyanobacteria* and *Planctomycetes* at the 2 -m depth and *Chloroflexi* at both depths required additional enzymatic lysis for improved OTU recovery. We also note that *Firmicutes* at 2 m produced no reads, regardless of permeabilization. Because of the low OTU recovery with bulk sequencing of this group, we suspect that this was a result of sampling bias rather than actual resistance of this phylum to epicPCR. We hypothesize that the *Proteobacterial* and *Cyanobacterial* OTUs at 2 m that were present in epicPCR experiments but not in bulk 16S sequencing result from the lower coverage of the bulk 16S sequencing.

Polyacrylamide formation and thermal cycling with additional enzymatic lysis proved sufficient to reproducibly recover rare candidate phyla, including H-178 with a 16S rRNA gene bulk read abundance of 7.8 × 10^−4^ (data not shown). epicPCR recovered this rare taxon using the nonspecific, barcode-16S assay design. Thus, the targeted, functional fusion approach could selectively amplify rare phyla and species to a much greater proportion of the final sequence data.

### epicPCR links metabolic functions to known and putative hosts

We repeated the *dsrB*-16S fusion on a larger number of cells to profile the lake water sulfate-reducing community. To confirm that epicPCR targets a wide range of bacterial reducing *dsrB* genes, we tested the primers *in silico* to a database of known *dsrAB* genes (Müller *et al.*, 2015) and compared the epicPCR *dsrB*s to bulk *dsrB* sequences (Supplementary Figure S3B). *In silico* PCR confirms that epicPCR primers have a broad specificity across bacterial reductive *dsrB*s but do not amplify bacterial oxidative *dsrB*s or archaeal reductive *dsrB*s. We observe an overlap between the bulk and epicPCR *dsrB* sequences and conclude that epicPCR targets a wide variety of reductive *dsrB* sequences in the lake water belonging to the *Deltaproteobacterial dsrB* supercluster (Supplementary Figure S3B). We suspect that the few hits of epicPCR *dsrB*s to oxidative or archaeal *dsrB*s result from low phylogenetic information of the *dsrB* fragment rather than low specificity of the epicPCR primers.

**Figure 4:**
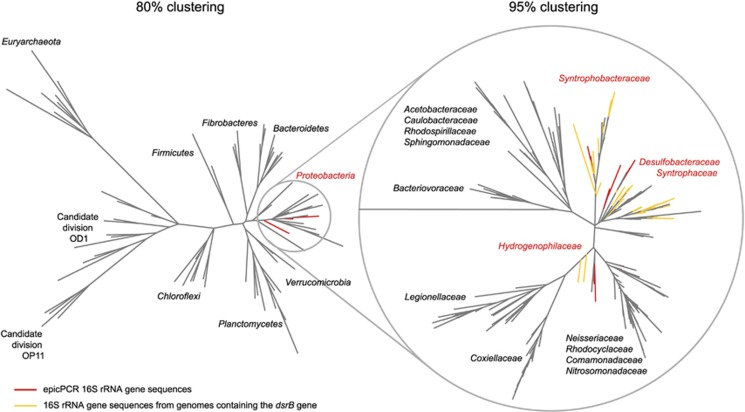
A maximum-likelihood tree of the microbial diversity in lake bottom water (21 m). The tree was constructed from the total 16 S rRNA gene sequences from lake bottom water clustered by 80% and 95% similarity, 16 S rRNA gene sequences belonging to known sulfate-reducing species (yellow branches) and 16 S rRNA gene sequences recovered by epicPCR by the presence of *dsrB* (red branches). The 16 S rRNA gene sequences recovered by epicPCR group within *Proteobacteria* with members from families *Desulfobacteraceae*, *Syntrophaceae*, *Syntrophobacteraceae*, which have previously been confirmed to contain the reductive *dsrB* gene (Muyzer and Stams, 2008).

From the same set of *dsrB*-16S fusion sequences, we analyzed the 16S rRNA genes to test whether our observations include known sulfate-reducing bacteria. A maximum- likelihood analysis (FastTree 2; Price *et al.*, 2010) grouped the epicPCR 16S rRNA gene sequences within the *Deltaproteobacterial* families *Syntrophobacteraceae*, *Syntrophaceae* and *Desulfobacteraceae* (Figure 4), members of which have been confirmed to contain the *dsrB* gene (Muyzer and Stams, 2008). Phylogenetic analysis against a database of known sulfate-reducing bacteria (Müller *et al.*, 2015) revealed that 319 364 out of 2 028 199 sequenced amplicons have <95% similarity to the closest known sulfate reducer and thus represent novel OTUs. Both novel and non-novel OTUs primarily have their closest matches in the Greengenes database to *Deltaproteobacteria* (Supplementary Table S6), indicating that novel groups found by epicPCR are likely not false positives. We also detect a fraction of 0.2% of *Gammaproteobacterial* and *Betaproteobacterial* reads that are most likely an unspecific background of the method.

## Discussion

Keeping pace with sequencing improvements, 16S rRNA gene and metagenomic surveys are now being enriched with methods to separate and characterize the function of single cells within the complex populations. Here we describe epicPCR, a novel technique to connect microbial function to phylogeny in a simple, high-throughput protocol. Using the highly parallel nature of emulsions, epicPCR provides a throughput of millions of cells with the cost of a single sequencing library preparation. We confirm the high specificity of epicPCR using synthetic control beads, and then successfully enrich for a collection of sulfate-reducing prokaryotes in the anoxic region of a stratified lake.

Key technological advances that are critical for an optimal performance of epicPCR include hydrogel formation and re-emulsification for fusion PCR, and certain optimizations for bulk downstream amplification. Sufficient dilution of cells or hydrogel beads prevents emulsion overloading, and adding glass beads into the tube during secondary emulsion production provides additional shear force to separate hydrogel beads into individual droplets (see Supplementary Methods). A three-primer fusion design ensures that only droplets containing a target gene produce amplicons, reducing unwanted 16S rRNA gene artifacts in the bulk mixture. Blocking primers, with highly efficient 3′ 3-carbon spacer blocks, also inhibit the spurious, chimeric amplification of incomplete fusion products within bulk reactions (Wetmur *et al.*, 2005; Turchaninova *et al.*, 2013).

epicPCR can determine the hosts of any target gene with conserved priming sites, and extensions of the method could generate quantitative or novel co-occurrence data. Our primer design only captured a small fraction of the *dsrB* gene, but an updated design could capture a long enough region of the target gene to construct dual target gene and 16S rRNA gene phylogenies to demonstrate coevolution or ancient horizontal transfers. Owing to the nonlinear effects of amplification and droplet size, the generated data forms a qualitative list of species rather than quantitative ratios. We expect that controlling droplet size with microfluidic droplet makers or tagging droplet products with molecular barcodes could produce quantitative results.

A variety of ecological questions become accessible with epicPCR, including which species drive biogeochemical cycles, harbor integrated phage or carry antibiotic resistance genes. Although these topics would require dispersing cell aggregates into single cells (as described in Kallmeyer *et al.* (2008) and Liu *et al.* (2011)), we also envision adaptations of epicPCR that would target more than one genome. epicPCR could query for host associations such as microbe–protist interactions by fusing 16S and 18S rRNA genes. By fusing a random barcode with 16S rRNA genes when targeting cell aggregates, fused 16S rRNA gene sequences under a single barcode would indicate physical co-occurrence and therefore spatial structuring of bacteria.

More general physical co-occurrence data could be collected by concatenating targets beyond bacterial genomic DNA. Combining the epicPCR concept with cDNA synthesis, the technique could have applications in immunology, including assaying the co-occurrence of T-cell receptor variable regions and T-cell master regulators (Turchaninova *et al.*, 2013). Extending from our current protocol, attachment of different functional molecules such as PCR primers or antibodies to the hydrogel matrix could lead to completely novel experimental strategies.
