# Supplementary material for: Massively parallel sequencing of single cells by epicPCR links functional genes with phylogenetic markers
Source: ISME J. 2015 Sep 22;10(2):427–36. doi: 10.1038/ismej.2015.124 (PMC4737934; doi:10.1038/ismej.2015.124)
Supplement: Supplementary Figure S1 [file ismej2015124x1.pdf]

Massively parallel sequencing of single cells by epicPCR links functional genes with phylogenetic markers

Supplementary Information

Sarah J. Spencer[1*], Manu V. Tamminen[2,3*], Sarah P. Preheim[2], Mira T. Guo[4], Adrian W. Briggs[5], Ilana L. Brito[2], Leena K. Pitkänen[3], Francois Vigneault[5], Marko P. Virta[3], Eric J. Alm[1,2,6,7,8]

[1] Computational and Systems Biology, Massachusetts Institute of Technology, Cambridge, MA 02139, USA
[2] Department of Biological Engineering, Massachusetts Institute of Technology, Cambridge, MA 02139, USA
[3] Department of Food and Environmental Sciences, University of Helsinki, Helsinki, Finland
[4] School of Engineering and Applied Sciences, Harvard University, Cambridge, MA 02138, USA
[5] AbVitro, Inc., Boston, MA 02210, USA
[6] Department of Civil and Environmental Engineering, Massachusetts Institute of Technology, Cambridge, MA 02139, USA
[7] The Center for Microbiome Informatics and Therapeutics, Massachusetts Institute of Technology, Cambridge, MA 02139, USA
[8] The Broad Institute of MIT and Harvard, Cambridge, MA 02139, USA
* These authors contributed equally to this work

Correspondence: EJ Alm, Department of Biological Engineering, Massachusetts Institute of Technology, 77 Massachusetts Avenue, Cambridge, MA 02139, USA. E-mail: ejalm@mit.edu; MV Tamminen, Department of Food and Environmental Sciences, University of Helsinki, PO Box 56, Helsinki, Finland. E-mail: mvtammin@mit.edu

Supplementary Methods……………………………………………………. 1
- epicPCR Reagents…………………………………………………… 1
- epicPCR Equipment…………………………………………………. 1
- epicPCR Solutions…………………………………………………… 1
- epicPCR Procedure………………………………………………….. 2
- epicPCR Accessory Procedures……………………………………… 4
- Sample collection, bulk 16S rRNA gene and *dsrB* gene library preparation…………………………………..………………6

Supplementary Figures……………………………………………………9

Supplementary Tables……………………………………………………..15

Supplementary References……………………………………………… 21

Supplementary Methods

epicPCR Reagents *(in addition to solution reagents)*
Ammonium persulfate (for molecular biology, ≥98.0%, Sigma, St. Louis, MO, USA)
TEMED (N,N,N′,N′-Tetramethylethylenediamine, ≥99.5%, Sigma)
Diethyl ether (water-saturated, ≥99.5%, Sigma)
Ethyl acetate (water-saturated, ACS grade, ≥99.5%, BDH, Poole Dorset, UK)
Agencourt AMPure XP - PCR Purification (Beckman Coulter, Danvers, MA, USA)
Ethanol (200 proof, VWR, Radnor, PA, USA)
Ready-Lyse Lysozyme Solution (Epicentre, Madison, WI, USA)
Proteinase K from *Tritirachium album* (for molecular biology, Sigma)
BSA (molecular biology grade, NEB, Ipswich, MA, USA)
Tween 20 (for molecular biology, Sigma)
Deoxynucleotide (dNTP) Solution Mix (10 mM each, NEB)
Phusion Hot Start Flex DNA Polymerase (NEB)
Ethylenediaminetetraacetic acid (EDTA, suitable for cell culture, Sigma)
SYBR Green I Nucleic Acid Gel Stain (10,000X, Invitrogen, Waltham, MA, USA)

epicPCR Equipment
1.5 ml Safe-Lock Microcentrifuge Tubes, Polypropylene (Eppendorf, Hamburg, DE)
2 ml Safe-Lock Microcentrifuge Tubes, Polypropylene (round-bottom, Eppendorf)
PCR 8-Well Tube Strips with Individually Attached Caps (VWR)
Microcentrifuge (Microcentrifuge 5415D, Eppendorf)
Thermal-cycler (C1000 Touch Thermal Cycler, Bio-Rad, Hercules, CA, USA)
BD Falcon 35µm Cell Strainer in 12x75 mm Polystyrene Tube (Corning, Tewksbury, MA, USA)
2 mm glass beads (Andwin Scientific, Schaumburg, IL, USA)
E-Gel iBase and E-Gel Safe Imager (Invitrogen)
E-Gel EX Agarose Gels, 1% (Invitrogen)

epicPCR Solutions
<u>Acrylamide solution</u> *(store at 4°C)*
12% Acrylamide (for molecular biology, ≥99.5%, Sigma)
0.32% BAC (N,N′-Bis(acryloyl)cystamine, suitable for electrophoresis, Sigma)

<u>1X TK buffer</u> *(store at RT)*
20 mM Tris-HCl (pH 7.5, Teknova, Hollister, CA, USA)
60 mM KCl (≥99.0%, VWR)

<u>STT emulsion oil</u> *(store at RT, should be prepared fresh every two weeks)*
4.5% Span 80 (Sigma)
0.4% Tween 80 (Sigma)
0.05% Triton X-100 (molecular biology grade, EMD Millipore, Billerica, MA, USA)
v/v in Mineral oil (light, suitable for cell culture, Sigma)

ABIL emulsion oil *(store at RT)*
4% ABIL EM 90, a surfactant (Evonik, Mobile, AL, USA)
0.05% Triton X-100 (molecular biology grade, EMD Millipore)
v/v in Mineral oil (light, suitable for cell culture, Sigma)

epicPCR Procedure

Polyacrylamide bead formation

To prepare polyacrylamide beads containing either cells or acrydited control molecules, we modified a polymerization protocol from (Tamminen and Virta, 2015). This involved the preparation of an aqueous suspension and then emulsification in an oil-surfactant solution. The 255 µl aqueous suspension included suspended cells or acrydited molecules, 0.98% ammonium persulfate (25 µl 10% APS), 9.4% acrylamide and 0.25% BAC (200 µl acrylamide solution). This suspension was applied to 600 µl STT emulsion oil, which was inverted and well-mixed before use, in a 2 ml round-bottom microcentrifuge tube and then vortexed for 30 s at 3000 rpm. We added TEMED to an aqueous concentration of 8.9% (25 µl TEMED) to catalyze the polymerization and vortexed for an additional 30 s at 3000 rpm, then let the emulsion polymerize for 90 min. Polyacrylamide beads were extracted with diethyl ether as described below, then filtered through a 35 µm cell strainer and transferred to a 1.5 ml microcentrifuge tube. Filtered polyacrylamide beads were stored at 4 °C and resuspended before subsequent lysis (described in main text).

Diethyl ether extraction for Span 80/Tween 80/Triton X-100 emulsions

When we phase-separated the emulsion oil from the polyacrylamide beads, we used an extraction protocol adapted from (Williams *et al.*, 2006). We added 800 µl of diethyl ether (the upper layer of water-saturated mixture) to each round-bottom tube containing an emulsion, then immediately flicked and inverted the tubes to mix the emulsions with the ether in order to form a visible precipitate. The ether/oil mixture surrounding the precipitate was discarded and replaced with 1 ml nuclease-free water, followed by mixing and inversion of the tubes.

Samples were transferred to standard microcentrifuge tubes and centrifuged for 30 s at 12,000 *g*. We observed three layers form: a bottom layer of polyacrylamide beads, a middle cloudy layer of oil/water, and a top milky layer of oil. The top oil layer was removed and discarded without disturbing the lower layer of polyacrylamide beads, then additional nuclease-free water was added and polyacrylamide beads were resuspended by flicking and inversion. The centrifugation, oil removal, and wash steps were repeated until there was no remaining oil forming an upper phase (approximately five washes). After the final wash, all the water was removed from the beads and beads were resuspended in 1 ml 1X TK buffer.

Emulsion-concatenation library preparation

To form initial fusion products, we combined a PCR mix with polyacrylamide bead templates and added the suspension to ABIL emulsion oil, which is more thermostable than STT oil (Williams *et al.*, 2006). The 100 µl PCR mix included 45 µl of polyacrylamide beads combined with PCR reagents and emulsion stabilizers (1X Phusion HF buffer, 1 mM $MgCl^2$, 250 µM each dNTP, 50 ng/µl BSA, 0.2% (v/v) Tween 20, and 0.16 U/µl Phusion Hot Start Flex). Additional primers and polyacrylamide beads used for specific samples are specified in Table S2, with sequences in Table S3. This mixture was placed in a 2 ml round-bottom microcentrifuge tube along with 900 µl ABIL emulsion oil. We also added four 2 mm autoclaved glass beads to the

emulsion components in order to promote polyacrylamide bead separation during the emulsification process. The oil and aqueous phases were vortexed at 3000 rpm for 1 min, then aliquot into PCR tubes for thermocycling (94 °C 30 s; 33 cycles of 94 °C 5 s, 52 °C 30 s, 72 °C 30 s; 72 °C 5 min; 10 °C hold). Following amplification, the aliquots from each sample were pooled, supplemented with 1 mM EDTA, extracted with diethyl ether and purified with a modified AMPure XP protocol as described below.

Following the initial fusion reaction, we nested within the fusion products for increased specificity in the final library. We used a standard PCR mix (1X Phusion HF Buffer, 200 μM each dNTP, 0.02 U/μl Phusion Hot Start Flex) and prepared four replicate 25 μl reactions for each sample. The reagents were combined with nested primers (Table S2, Table S4), blocking primers (3.2 μM U519F_block10, 3.2 μM U519R_block10), and 2-5 μl of purified product from the previous fusion reaction. The thermocycling program (98 °C 30 s; 40 cycles of 98 °C 5 s, 52 °C 30 s, 72 °C 30 s; 72 °C 5 min; 10 °C hold) contained 40 PCR cycles by default. We reduced the number of cycles for the nested reaction whenever possible based on qPCR Ct values collected prior to the final nested reaction; these Ct values were collected using the same reaction conditions plus 0.5X SYBR Green I. Following amplification of the final nested reactions, the four replicate reactions were pooled and purified according to the modified AMPure XP protocol below.

The fused, nested products underwent a final, short amplification with Illumina adapters, then samples were pooled and submitted for sequencing. For each sample, we first assembled four replicate reactions using the standard Phusion Hot Start Flex reaction conditions. In the replicate reactions for a single sample we used 3.3 μM PE-PCR-F plus 3.3 μM PE-PCR-XXX to serve as a sample barcode (Table S5). We amplified the libraries (98 °C 30 s; 7 cycles of 98 °C 30 s, 83 °C 30 s, 72 °C 30 s; 10 °C hold) and then pooled replicate reactions and purified with AMPure XP beads according to the modified protocol below. The appropriate amplicon size was confirmed on a 1% agarose E-Gel according to the manufacturer's instructions. Barcoded sample libraries were pooled in equal stoichiometric ratios and sequenced on an Illumina MiSeq with 20% phi-X spike-in to provide template diversity. We sequenced paired-end libraries with 250 bp reads in both directions and an 8 bp sample barcode read.

Diethyl ether extraction for ABIL EM 90/Triton X-100 emulsions

For the phase-separation of soluble fusion products from ABIL EM 90 oil emulsions, we again adapted a protocol from (Williams *et al.*, 2006). Each sample was pooled and centrifuged at 13,000 *g* for 5 min at 25 °C. The upper (oil) phase was discarded and replaced with 1 ml diethyl ether (upper layer of water-saturated mixture), then vortexed to mix. Samples were centrifuged for 1 min at 13,000 *g* to separate the phases so that the upper phase could be discarded. This ether wash was repeated, then the same extraction was performed with ethyl acetate (upper layer of water-saturated mixture). We performed two more extractions with diethyl ether, then disposed of the upper phase. Samples were left open in a chemical hood for 10 min so the remaining diethyl ether could evaporate. For each sample we recovered 100-150 μl from the bottom phase into a fresh 1.5 ml microcentrifuge tube for purification prior to the nested PCR.

Modified AMPure XP purification

Our approach follows the manufacturer's protocol with the following variations. The AMPure XP beads were always equilibrated to room temperature (~30 min) before use. The beads were added to 1.5 ml microcentrifuge tubes in a ratio of 0.9 μl AMPure XP beads per 1 μl of PCR

product. All mixing steps were completed by gentle vortexing or flicking. Upon addition of the AMPure XP beads, the solution was mixed and incubated for 13 min at room temperature. Two ethanol washes following magnetic separation were performed with 500 µl of 70% EtOH, and then the beads were air-dried for 15-20 min. The elution buffer (Buffer EB, Qiagen, Venlo, NL) was incubated with the beads for 7 min, then tubes were placed on a magnet for 2 min. The eluate was collected and transferred to a fresh tube.

epicPCR Accessory Procedures

Preparation of synthetic control polyacrylamide beads

In order to produce 348 bp segments of acrydited DNA sequence to incorporate into our positive and negative control polyacrylamide beads, we synthesized the sequences without the modification and then added the acrydite modification via PCR. The un-modified template DNA sequences (16S-V4neg and 16S-V4pos, Table S1) were amplified in five replicate reactions using Phusion Hot Start Flex DNA Polymerase (NEB). The 50 µl reaction conditions were composed according to the manufacturer's protocol and included 0.5 µM each of 16S-synthF and 16S-synthR, along with 10 ng/reaction of un-modified template DNA (Table S1). We cycled with standard conditions (98 °C 30 s; 25 cycles of 98 °C 5 s, 66 °C 30 s, 72 °C 30 s; 72 °C 10 min; 10 °C hold), then pooled the replicate reactions and purified with the MinElute PCR Purification Kit (Qiagen). The modified 16S-V4neg and 16S-V4pos sequences were used along with the independently synthesized dsrB-synth to attach to polyacrylamide control beads. The attachment was accomplished by mixing these acrydited amplicons with acrylamide solution and polymerizing as described in 'Polyacrylamide bead formation'.

Parallel epicPCR assay for rare target genes

In order to assay an increased number of cells and comprehensively sequence the species carrying *dsrB*, we performed the 21 m *dsrB*-16S rRNA gene fusion (abbreviated *dsrB*-16S) in multiple emulsion tubes and then combined the fusion products. Using previously polymerized polyacrylamide bead templates, we completed ten emulsion-concatenation reactions as described above and then combined the recovered aqueous phases. To purify and concentrate the fusion products, we used a MinElute PCR purification kit (Qiagen) instead of AMPure XP beads, concentrating ~1500 µl of recovered aqueous phase into a 10 µl final eluate. The concentrated fusion products were amplified with our nested PCR design for 40 thermal cycles, then labeled with a single sample barcode and flanked with Illumina paired-end sequencing adapters as described above. We loaded the final library on a 1% agarose E-Gel and excised the library band for purification using the Qiagen Gel Extraction Kit. Sequencing this library produced high-quality, paired-end fusion reads that matched our primer design and contributed to Figure 4.

Emulsion microscopy

In order to visualize emulsion droplets, both with and without polyacrylamide beads, we pipette dilute emulsions into a hemacytometer (Bright Line Counting Chamber, Hausser Scientific, Horsham, PA, USA). We combined 1 µl emulsion droplets with 9 µl mineral oil in a fresh microcentrifuge tube. This dilute emulsion was loaded into the hemacytometer and viewed at 100X resolution. We used the hemacytometer rulings to spot check the average droplet size of primary emulsions and also to quantify polyacrylamide bead loading in the secondary emulsion. In the secondary emulsion, out of nine 4000 μm^2 hemacytometer sections we observed 275 normal droplets and 4 droplets with two or more polyacrylamide beads. With our positive and

negative spike-in ratios of approx. 2,000 control beads per 22,000,000 total beads, we expected and observed no negative fusion products owing to a 90% ratio of empty environmental beads. Fluorescence images presented in Figure S1 were generated according to the SYBR Green I manufacturer's protocol.

dsrB primer design

We designed primers to target the beta subunit of dissimilatory sulfite reductase (*dsrB*) by using gene alignments and adapting primers from (Wagner *et al.*, 1998) and (Giloteaux *et al.*, 2010). Our dsrB-F1 primer (Table S3) is equivalent to the dsr4R primer in (Wagner *et al.*, 1998). Our bridge primer, dsrB-R1_519R, contains a *dsrB* priming sequence based on the 1905 priming site in (Giloteaux *et al.*, 2010), but shifted over nine positions to fall at position 1896 of the *Desulfovibrio vulgaris* gene. It also contains added ambiguities: C→Y in position 6 of 1905 and C→Y in position 9 of 1905. Finally, our i_dsrB-F3 nested primer is the reverse complement of 1929 with additional ambiguities Y→S in position 12 and Y→B in position 15 (Giloteaux *et al.*, 2010).

epicPCR sequence analysis and OTU clustering

For data analysis, we used the QIIME package with a few additional custom python scripts. To join the paired-end forward and reverse reads, we ran the QIIME command join-paired_ends.py with default parameters. The samples were demultiplexed and quality filtered with the QIIME command split_libraries_fastq.py (--min_per_read_length_fraction 0.40 -q 20 --max_barcode_errors 0 --max_bad_run_length 0). Following chimera identification using identify_chimeric_seqs.py (-m usearch61), we discarded chimeric sequences with a customized python script (version 2.7; https://github.com/sjspence/epicPCR/blob/master/discardChimeras.py). The remaining reads were filtered by length and expected fusion structure using custom python scripts. Our structure-filtering python scripts discarded any sequences that did not carry the expected forward, reverse, and bridge primers, then exported 121 bp of the captured 16S rRNA gene V4 variable region (version 2.7; https://github.com/sjspence/epicPCR/blob/master/filter*.py). If barcoded reads shared an identical droplet barcode and identical 16S rRNA gene sequence, we collapsed them into a single representative sequence using https://github.com/sjspence/epicPCR/blob/master/compressBar.py.

Our BLAST analysis for negative and positive control sequences relied on a simplified, custom BLAST database search. We used the synthetic designed sequences (16S_V4neg and 16S_V4pos, Table S1) as a two-item database for our filtered 16S rRNA gene V4 fusion Illumina reads. The blastall 2.2.22 tool with default parameters identified reads with a significant match to our synthetic sequences (Vakatov, 2013). Both of these synthetic 16S rRNA gene V4 sequences were generated randomly and were thus highly divergent from any evolved 16S rRNA gene sequences.

For Operational Taxonomic Unit (OTU) assignment, we again relied on QIIME functions using default parameters unless otherwise specified. Starting from our stitched, quality-filtered, structure-filtered, length-trimmed 16S rRNA gene V4 sequences, we ran a series of commands to group and classify 97% identity sequence clusters. Our commands included pick_otus.py, pick_rep_set.py (-m most_abundant), assign_taxonomy.py, make_otu_table.py, and summarize_taxa.py. For datasets that compared multiple samples (e.g. Fig. 3), we rarefied the 16S rRNA gene V4 reads to the sample with the lowest read count using custom scripts in R. This rarefaction was performed after forming individual OTU tables but before summarizing

taxonomic abundances. Computational commands are also presented step-by-step in a README file at https://github.com/sjspence/epicPCR/blob/master/README.md.

Sample collection, bulk 16S rRNA gene and *dsrB* gene library preparation

Sample collection

Water was collected from Upper Mystic Lake, (Winchester, MA, ~ 42 26.155N, 71 08. 961W) on Aug. 12, 2013 using a peristaltic pump and plastic Tygon tubing. Ethanol was applied to the end of the tubing and gloves were worn during collection to prevent contamination of samples during collection. A Hydrolab minisonde (Hach Hydromet, Loveland, CO, USA) attached to the end of the tubing recorded depth, dissolved oxygen, temperature, pH and specific conductance during deployment. Water from depth was allowed to flow through the tubing for 2 volumes (2 L) before 50 ml of water was filtered through a 0.22 µM filter in a 25 mm Swinnex-25 Filter Holder (Millipore, Darmstadt, DE) for DNA extraction. Filters were placed in a plastic bag and immediately placed on dry ice. For epicPCR, 7 ml of water was also added to 7 ml of 50% sterile glycerol in a 15 ml conical tube and immediately placed on dry ice. In parallel, aliquots were collected for nitrate and sulfate measurements via Ion Chromatography at the University of New Hampshire Water Resources Research Center. Blanks were collected by pumping 2 L of sterile water through the tubing before and after sampling to determine the influence of both contamination from the tubing and sampling method as well as carryover from the previous sample.

Bulk DNA extraction

Filters were stored at -80 °C until extraction. DNA was extracted from the filters using PowerWater DNA extraction kit (Mo Bio, Carlsbad, CA, USA) with an alternative lysis and proteinase K incubation step. Filters were removed from filter holders in a laminar flow hood and placed into the PowerWater Bead tube with a pair of sterile forceps as recommended. 1 ml of PW1 was added to the Bead tube, along with 20 µl of proteinase K (>600 mAU/ml, Qiagen). The alternative lysis protocol was followed by incubating samples at 65 °C for 10 minutes. Following the alternative lysis, the PowerWater protocol was followed, including horizontal vortexing with the recommended Mo Bio vortex adapter for 5 minutes and all subsequent steps. Purified DNA was stored at -20 °C.

Illumina 16S rRNA gene library preparation

The 16S rRNA gene libraries were prepared as previously described (Preheim *et al.*, 2013). Briefly, real-time PCRs were done first to normalize template concentrations and avoid cycling any templates past mid-log phase. PCRs for Illumina libraries were carried out as follows: 0.5 units of Phusion with 1X High Fidelity buffer, 200 µM of each dNTP, 0.3 µM of PE16S_V4_U515_F (5'-ACACGACGCTCTTCCGATCTYRYRGTGCCAGCMGCCGCGGTAA-3') and PE16S_V4_E786_R (5'-CGGCATTCCTGCTGAACCGCTCTTCCGATCTGGACTACHVGGGTWTCTAAT-3') first step primers and approximately 40 ng of mixed DNA template were added for each 25 µl reaction. Additionally, 5X SYBR Green I nucleic acid stain (Molecular Probes, Eugene, OR, USA) was added for real-time PCR. Samples were cycled with the following conditions: denaturation at 98 °C for 30 s, annealing at 52 °C for 30 s, and extension at 72 °C for 30 s. Samples were normalized to 20 cycles with the following dilution: $1.75^{(Ct-20)}$ or undiluted for samples with Ct larger than 20. The first step PCR was cycled as four

25 µl reactions for each sample with 20 cycles of amplification. PCRs were pooled and cleaned with Agencourt AMPure XP-PCR purification (Beckman Coulter) according to 'Modified AMPure XP purification' described above. Illumina-specific adaptors were added during a second step amplification, which include the sample specific barcode (index) sequences (Table S5). The conditions for the second step PCR were similar to the first step, although 4 µl of the purified first step reaction was used as a template and 0.4 µM of each PE-PCR-F and the barcoded reverse primer was used with 9 cycles. Samples were cycled as four 25 µl reactions and cleaned with the Agencourt AMPure XP-PCR purification system using a modified protocol described above. Six samples (three samples, three blanks and three controls) were sequenced across 3 different MiSeq runs with multiple other samples.

Illumina dsrB gene library preparation

We amplified a region of the *dsrB* gene from bulk genomic DNA in order to compare the bulk *dsrB* diversity with epicPCR gene fusions. A 1:5 dilution of genomic DNA recovered from the bulk DNA extraction served as template for amplification with primers i_DSR1097AF (5'-CGGCATTCCTGCTGAACCGCTCTTCCGATCTGGAHTKGTGGATGGAAGA-3') and i_dsrB-F1 (5'-ACACGACGCTCTTCCGATCTYRYRGTGTAGCAGTTACCGCA-3'). We sourced the primer DSR1097AR from Giloteaux *et al.*, reverse complemented it (underlined), and added an Illumina adapter to produce i_DSR1097AF. From the same study we sourced DSR4R (underlined) and simply added an Illumina adapter to produce i_dsrB-F1 (Giloteaux *et al.*, 2010). For each of six samples, we prepared quadruplicate 25 µl PCRs (1X Phusion HF Buffer, 200 µM dNTPs, 0.5 µM i_DSR1097AF, 0.5 µM i_dsrB-F1, 0.5 U Phusion Hot Start Flex DNA Polymerase, 2 µl 1:5 genomic template). After cycling (94 °C 30 s; 25 cycles of 94 °C 5 s, 52 °C 30 s, 72 °C 30 s; 4 °C hold), quadruplicate reactions from each sample were pooled and purified with the Agencourt AMPure XP-PCR purification system using a modified protocol described above.

These six purified libraries were amplified again to add final Illumina adapters and barcodes. Each sample was amplified in quadruplicate 25 µl PCRs (1X Phusion HF Buffer, 200 µM dNTPs, 0.4 µM PE-PCR-F, 0.4 µM PE-PCR-XXX, 0.5 U Phusion Hot Start Flex DNA Polymerase, 4 µl purified reaction from previous step). After cycling (98 °C 30 s; 15 cycles of 98 °C 10 s, 83 °C 30 s, 72 °C 60 s; 4 °C hold), quadruplicate reactions from each sample were pooled. Three samples were purified with the Agencourt AMPure XP-PCR purification system using a modified protocol described above. The other three were gel-purified to select for an 1,036 bp insert size which corresponds to the majority of published *dsrB* variants. The three non-gel purified and three gel-purified sample libraries were pooled in equal stoichiometric ratios and sequenced on an Illumina MiSeq with 20% phi-X spike-in to provide template diversity. We sequenced paired-end libraries with 250 bp reads in both directions and an 8 bp sample barcode read.

Bulk 16S rRNA gene sequence data processing

Paired end sequence data from each run was processed with SHERA (Rodrigue *et al.*, 2010), filtering out overlaps with less than 80% confidence (filterReads.pl with 0.8). Sequence and quality files were merged into fastq format with mothur make.fastq (Schloss *et al.*, 2009). Resulting fastq files were quality filtered and demultiplexed with QIIME split_libraries_fastq.py with the following options: truncate at positions in the read with quality scores less than 10 using ascii offset of 33 (-q 10 --max_bad_run_length 0 --phred_offset 33) and remove resulting reads

shorter than 80% of the read length (-min_per_read_length .8). Primers were removed with a custom perl script, searching for the primer sequence 9 bp from either end of the forward and reverse position and allowing 4 bases of ambiguity at the end of the primer for mismatch repair. Processed reads were trimmed to 121 bp with a custom python script (version 2.7; https://github.com/sjspence/epicPCR/blob/master/filterLength.py) and then classified into OTUs according to 'epicPCR sequence analysis and OTU clustering'.

Supplementary Figures

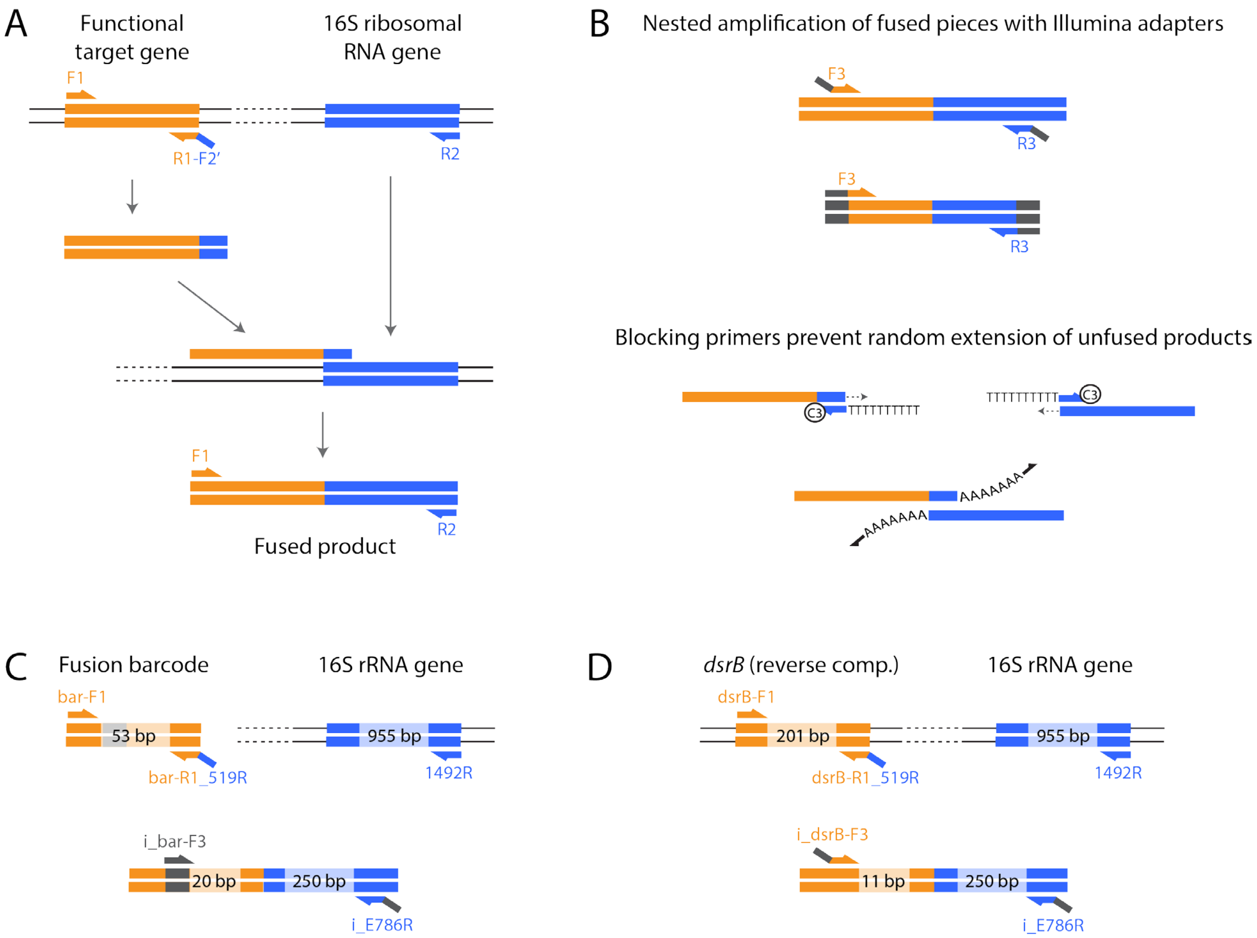

Figure S1. epicPCR primers fuse target genes within droplets and then enrich for successful fusion constructs in a bulk nested reaction. A) Fusion PCR joins together two amplicons in a single reaction. The amplification first proceeds exponentially for the functional target gene from primers F1 and R1-F2' and linearly for the 16S ribosomal RNA gene from primer R2. Primer R1-F2' adds an overhang to the target gene amplicon that is specific to the start of 16S ribosomal RNA gene. Primers F1 and R2 are in excess over R1-F2', causing its depletion during the early cycles of PCR. After depletion of R1-F2', the 16S ribosomal RNA-specific overhang of the target gene amplicon primes the 16S ribosomal RNA gene creating a fused product. This fused product is subsequently exponentially amplified by F1 and R2. B) In the nested reaction, successful fusion products are amplified with Illumina adapters while partial fusion products are dampened by blocking primers. The blocking primers, added in excess, anneal to the universal 519R sequence but do not extend from the primer end due to a 3' 3-carbon-spacer. Instead, extension occurs from the 3' end of partial fusion products into the overhang region of the blocking primer, adding a string of A bases to the partially fused pieces. This A tail prevents partially fused pieces from annealing, extending, and generating spurious fusion products. C) Fusion construct design for fusions between a soluble molecular barcode and the 16S rRNA gene. The first row shows the initial fusion design and the second row shows the nested reaction design. D) Fusion construct design for fusions between bacterial *dsrB* and the 16S rRNA gene.

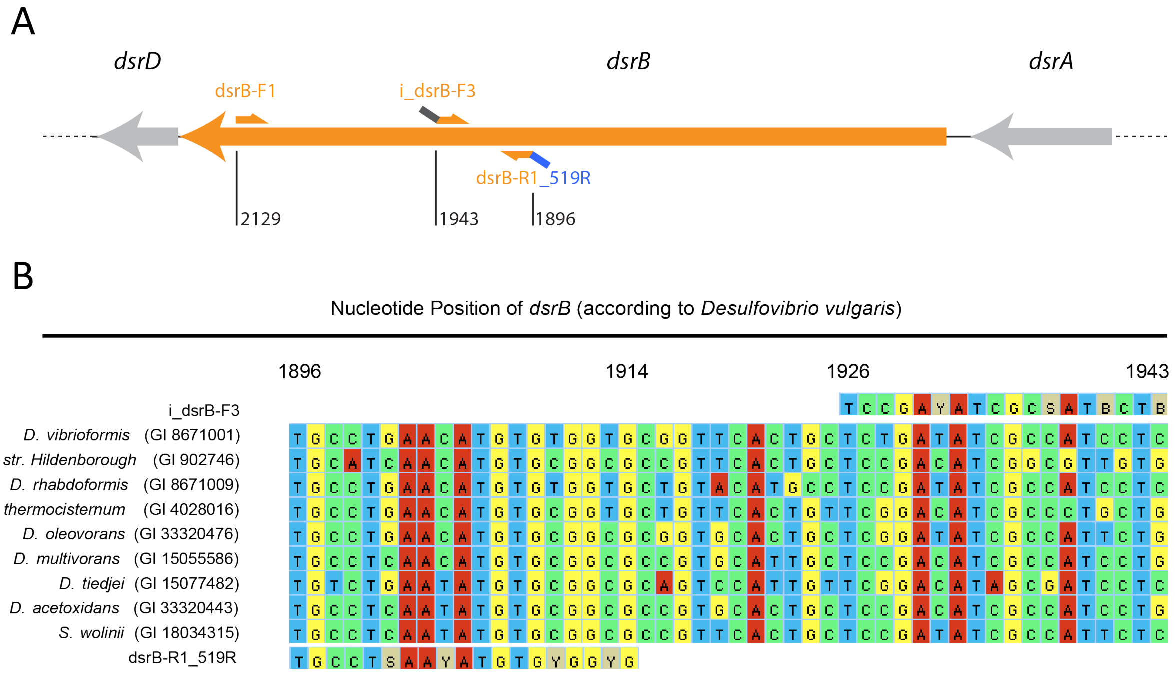

Figure S2. Degenerate primers target the *dsrB* gene for epicPCR. A) A schematic showing the three *dsrB* primers in their approximate genomic context. Nucleotide positions below the primers are based on the *Desulfovibrio vulgaris dsrB*. B) A selection of nucleotide alignments demonstrating the genomic context and selected degeneracies for primers i_dsrB-F3 and dsrB-R1_519R (Giloteaux *et al.*, 2010).

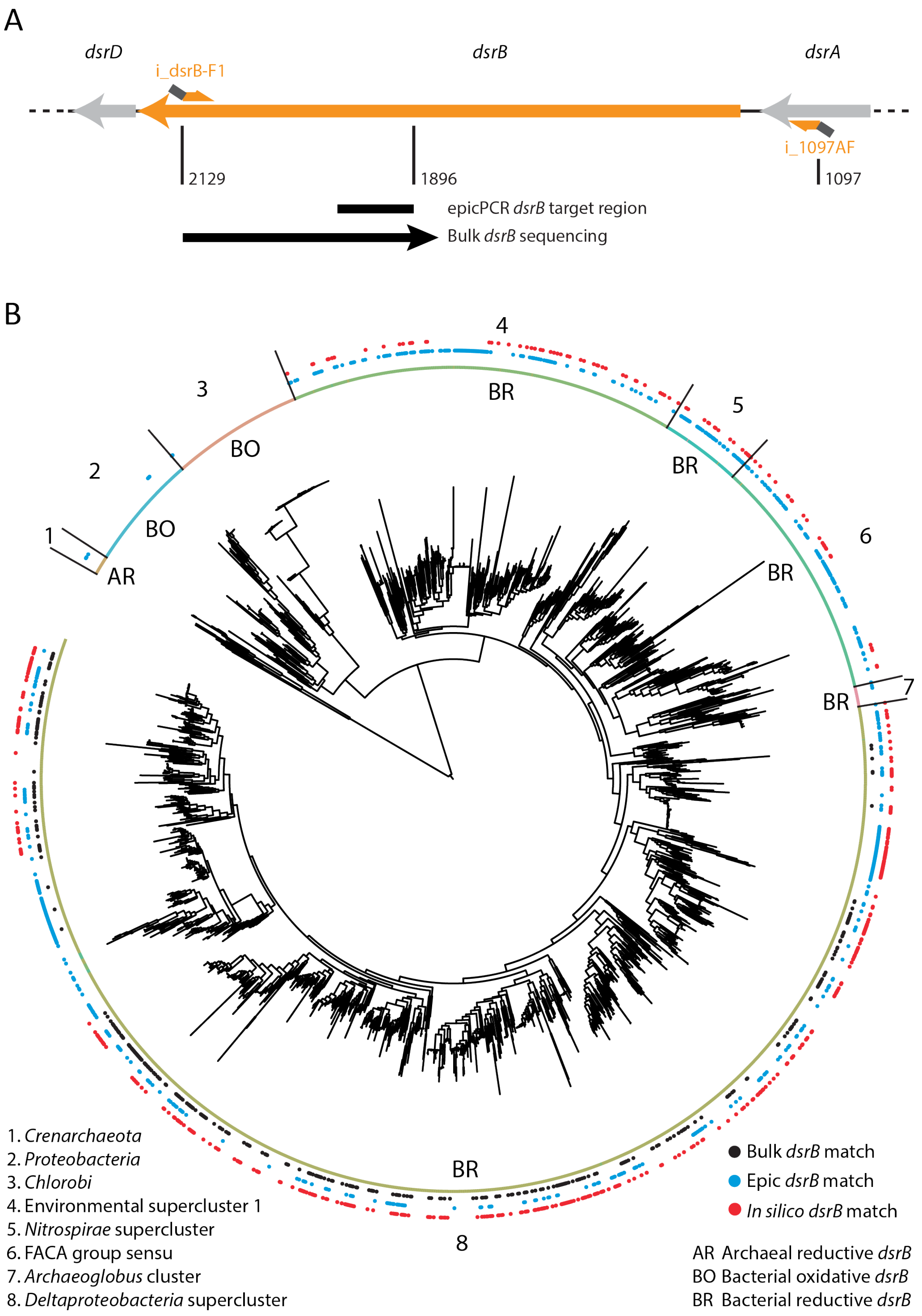

Figure S3. Bulk *dsrB* gene fragment short-read sequencing provides a background distribution for observed epicPCR *dsrB* fragments. A) A schematic showing the bulk *dsrB* primers, modified from (Giloteaux *et al.*, 2010), in their approximate genomic context. Nucleotide positions below the primers are based on the *Desulfovibrio vulgaris dsrB*. Grey primer overhangs indicate Illumina adapter sequences. B) Distribution of bulk *dsrB* sequencing reads, epicPCR *dsrB* reads and *in silico* epicPCR *dsrB* matches in a tree of known *dsrAB* genes (Müller *et al.*, 2015).

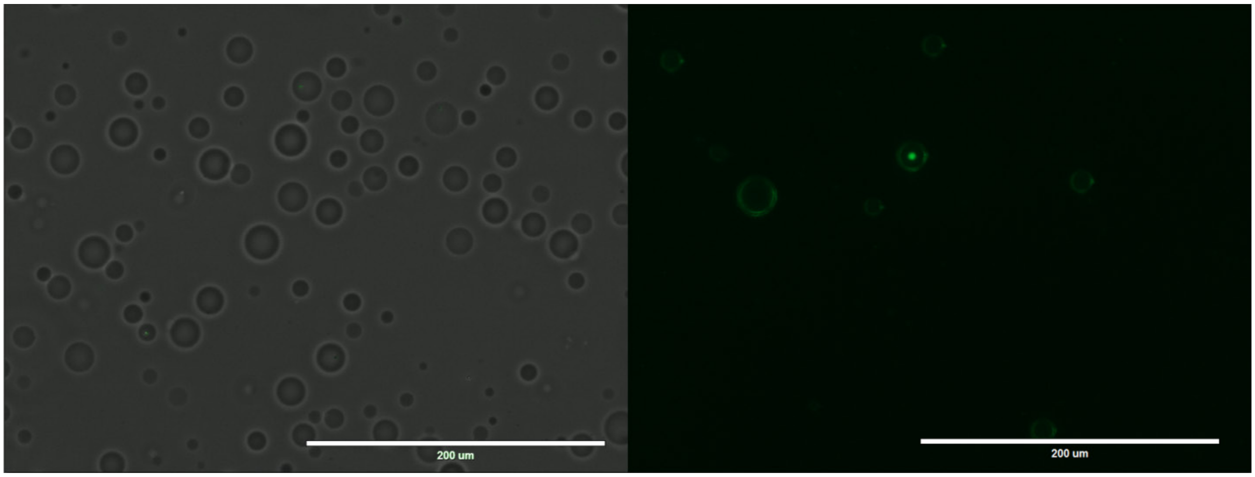

Figure S4. Vortex-generated emulsions separate single cells or single polyacrylamide beads into nanoliter volume droplets. A) Single cells disperse into individual droplets, with the majority of droplets empty. This merged image shows bright-field emulsion droplets overlaid with a fluorescence image of SYBR-stained bacterial cells. B) Polyacrylamide beads in the secondary emulsion carry bacterial chromosomes as templates for fusion PCR. This fluorescence image shows a SYBR-stained bacterial genome in a polyacrylamide bead, suspended in emulsion oil prior to fusion PCR.

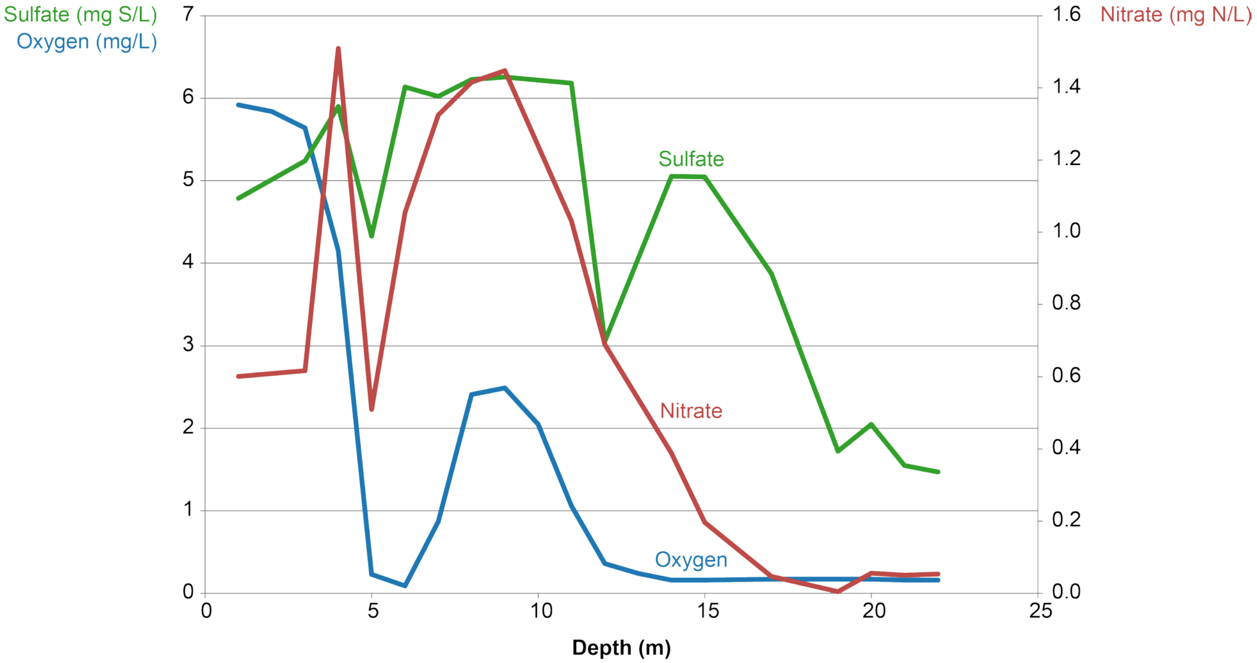

Figure S5. Observed geochemistry at different lake depths collected on 8/12/2013. At a 2 m depth, oxygen predominates. At a 21 m depth, both oxygen and nitrate are depleted, but sulfate is still available as an electron acceptor.

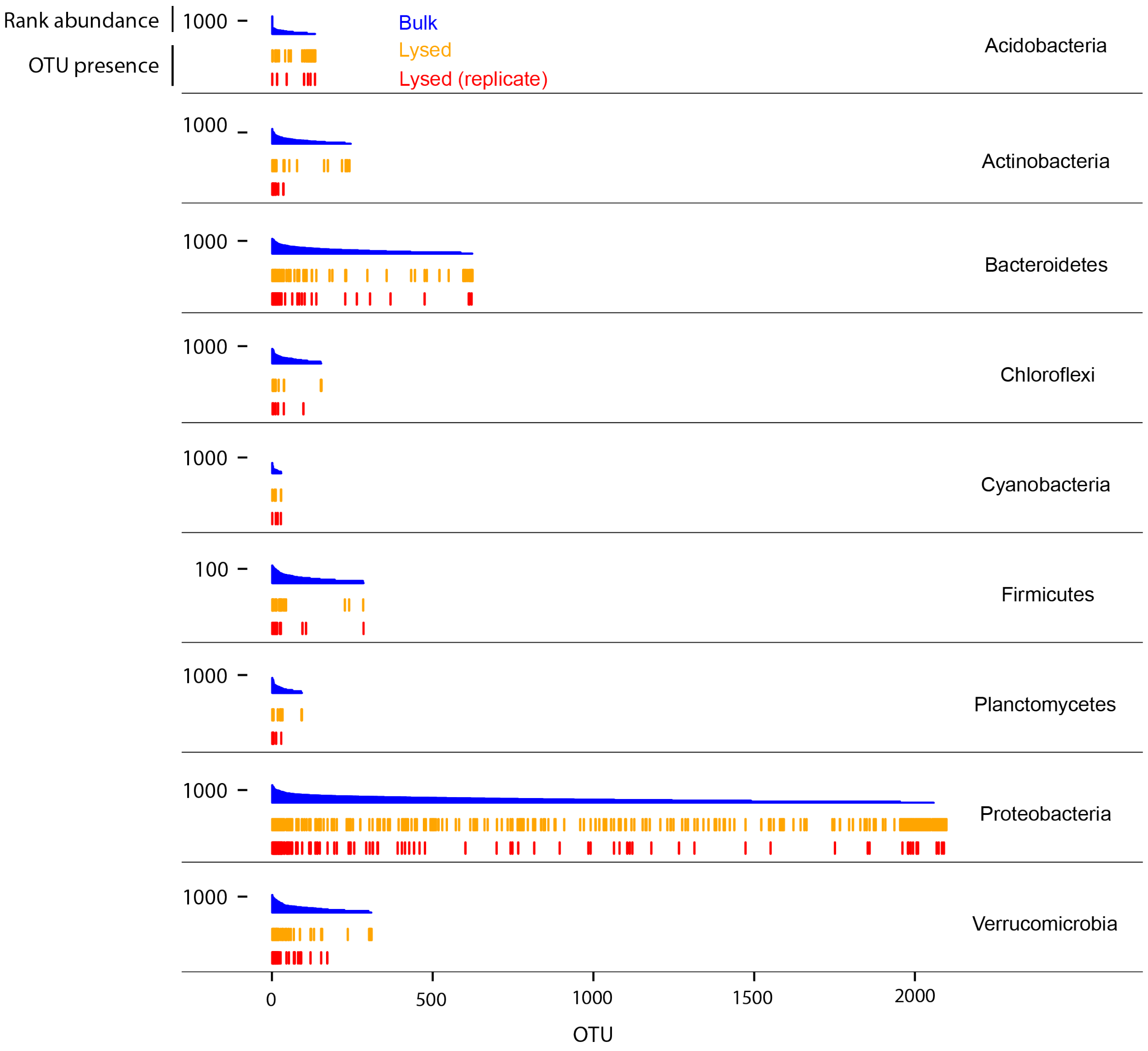

Figure S6. Duplicate epicPCR barcoded libraries from the 21 m lake depth. OTUs are listed by phyla according to the rank-ordered abundance from bulk 16S rRNA gene sequencing. Below the bulk sequencing, the presence of an OTU in duplicate, lysed 21 m epicPCR libraries is indicated by an orange or red bar.

Supplementary Tables

Table S1. Primers used for synthetic bead preparation. DNA was incorporated into polyacrylamide hydrogels via an acridite modification at the 5' end of the sequence (/5Acryd/). The synthetic *dsrB* sequence was synthesized directly with the acrydite attachment (dsrB-synth). The acrydite attachment was added to the synthetic 16S rRNA gene V4 sequences (16S-V4neg, 16S-V4pos) using an acrydited forward primer (16S-synthF) in a PCR (see Supplementary Methods). Colors indicate identical or reverse complement primers and corresponding priming sites.

**Sequence Name**	**Sequence (5' → 3')**
dsrB-synth	/5Acryd/GTGTAGCAGTTACCGCAGAGGATGGCGATATCGGAGCATTGCACCACACATGTTCAGGCA
16S-synthF	/5Acryd/**TCGAGGCCGTTCGTTAATTC**
16S-synthR	**GGAGCGTCCGGTATTGATTG**
16S-V4neg	**TCGAGGCCGTTCGTTAATTC**CAGCAGCCGCGGTAATACGTAAACTACGATGGCACCAACTCAATCGCAGCTCGTGCGCCCTGAATAACGTACTCATCTCAACTGATTCTCGGCAATCTACGGAGCGACTTGATTATCAACAGCTGTCTAGCAGTTCTAATCTTTTGCCAACATCGTAATAGCCTCCAAGAGATTGATCATACCTATCGGCACAGAAGTGACACGACGCCGATGGGTAGCGGACTTTTGGTCAACCACAATTCCCCAGGGGACAGGTCCTGCGGTGCGCATTAGATACCCTGGTAGTCCAAAGTCGTAACAAGGTAACC**CAATCAATACCGGACGCTCC**
16S-V4pos	**TCGAGGCCGTTCGTTAATTC**CAGCAGCCGCGGTAATACATAGCCGCGCTATCCGACAATCTCCAAATTATAACATACCGTTCCATGAAGGCCAGAATTACTTACCGGCCCTTTCCATGCGTGCGCCATACCCCCCCACTCCCCCGCTTATCCGTCCGAGGGGAGAGTGTGCGATCCTCCGTTAAGATATTCTTACGTATGACGTAGCTATGTATTTTGCAGAGGTAGCGAACGCGTTGAACACTTCACAGATGGTGGGGATTCGGGCAAAGGGCGTATAATTGGGGACATTAGATACCCTGGTAGTCCAAAGTCGTAACAAGGTAACC**CAATCAATACCGGACGCTCC**

Table S2. Primers used for epicPCR. We synthesized a fusion barcode with 20 degenerate bases that we spiked in at low concentrations in order to fuse it to any 16S rRNA genes available in each droplet. We performed barcode-16S rRNA gene fusions with bar-F1, 1492R, and bar-R1_519R as a bridge primer. We performed *dsrB*-16S fusions with dsrB-F1, 1492R, and dsrB-R1_519R as a bridge primer. Universal primer segments 1492R and 519R were drawn from (Lane, 1991) and *dsrB* primers were adapted from (Wagner *et al.*, 1998; Giloteaux *et al.*, 2010). Colors indicate identical or reverse complement sequences between Tables S3-S5.

**Primer Name**	**Sequence (5' → 3')**
fusionBarcode	CGGCACAATCTCGTCGCGTCGACACTCTTTCCCT**ACACGACGCTCTTCCGATCT**NNNNNNNNNNNNNNNNNNNNGATCATGACCCATTTGGAGAAGATG
bar-F1	CGGCACAATCTCGTCGCGTCG
1492R	GGTTACCTTGTTACGACTT
bar-R1_519R	**GWATTACCGCGGCKGCTG**CATCTTCTCCAAATGGGTCATGATC
dsrB-F1	GTGTAGCAGTTACCGCA
dsrB-R1_519R	**GWATTACCGCGGCKGCTG**TGCCTSAAYATGTGYGGYG

Table S3. Samples, conditions, and primer sets used to produce particular epicPCR libraries. At the 2 m depth, 7×10^7 cells were suspended in polyacrylamide beads. At the 21 m depth, 1.4×10^7 cells were suspended in polyacrylamide beads. Use of lysis reagents is described in the main text. When control beads were spiked in, we added 0.5 µl 200X dilution of the initial bead preparation as described in Supplementary Methods. Concentrations of listed primers in the final fusion reactions were 100 fM fusion barcode, 1 µM F1, 1 µM R2, and 10 nM R1-F2'. Concentrations of listed primers in the subsequent nested reactions were 0.3 µM Nested F3 and 0.3 µM Nested R3.

Depth	Lysis reagents	Control beads	Fusion barcode	F1	R2	R1-F2'	Nested F3	Nested R3
2 m	-	-	+	bar-F1	1492R	bar-R1_519R	i_bar-F3	i_E786R
2 m	+	-	+	bar-F1	1492R	bar-R1_519R	i_bar-F3	i_E786R
2 m	+	+	+	bar-F1	1492R	bar-R1_519R	i_bar-F3	i_E786R
21 m	-	-	+	bar-F1	1492R	bar-R1_519R	i_bar-F3	i_E786R
21 m	+	-	+	bar-F1	1492R	bar-R1_519R	i_bar-F3	i_E786R
21 m	+	+	+	bar-F1	1492R	bar-R1_519R	i_bar-F3	i_E786R
2 m	+	-	-	dsrB-F1	1492R	dsrB-R1_519R	i_dsrB-F3	i_E786R
2 m	+	+	-	dsrB-F1	1492R	dsrB-R1_519R	i_dsrB-F3	i_E786R
21 m	+	-	-	dsrB-F1	1492R	dsrB-R1_519R	i_dsrB-F3	i_E786R
21 m	+	+	-	dsrB-F1	1492R	dsrB-R1_519R	i_dsrB-F3	i_E786R
21 m	+	-	-	dsrB-F1	1492R	dsrB-R1_519R	i_dsrB-F3	i_E786R

Table S4. Primers used for the nested PCR. Either the i_bar-F3 or i_dsrB-F3 primers were used in the forward direction, paired with i_E786R in the reverse direction. The blue and red segments are overhangs used for Illumina adapter addition (see Table S5). The underlined segment of i_dsrB-F3 indicates a small degenerate sequence that was added to increase the sequence complexity of the amplicon library for improved Illumina image analysis. The blocking primers, U519R-block10 and U519F-block10, carry a 3-carbon spacer to prevent 3' extension; this forces the addition of A bases to the 3' end of any unfused pieces. Universal primer segments E786R and 519R/F were drawn from (Lane, 1991) and *dsrB* primers were adapted from (Wagner *et al.*, 2005). Colors indicate identical or reverse complement sequences between Tables S3-S5.

Primer Name	Sequence (5' → 3')
i_bar-F3	**ACACGACGCTCTTCCGATCT**
i_dsrB-F3	**ACACGACGCTCTTCCGATCT**YRYRVAGVATSGCGATRTCGGA
i_E786R	**CGGCATTCCTGCTGAACCGCTCTTCCGATCT**GGACTACHVG GGTWTCTAAT
U519R-block10	TTTTTTTTTT**GWATTACCGCGGCKGCTG**/3SpC3/
U519F-block10	TTTTTTTTTT**CAGCMGCCGCGGTAATWC**/3SpC3/

Table S5. Primers used for Illumina library preparation**.** The forward primer PE-PCR-F can pair with any of the reverse primers (PE-PCR-XXX). The numbered primer names indicate reverse primers with different Illumina barcode sequences that can serve as sample identifiers in pooled sequencing runs. The underlined sequence indicates the unique sample barcode within these reverse primers. Colors indicate identical or reverse complement sequences between Tables S3-S5.

Primer Name	Sequence (5’ → 3’)
PE-PCR-F	AATGATACGGCGACCACCGAGATCTACACTCTTTCCCT**ACACGACGCTCTTCCGATCT**
PE-PCR-001	CAAGCAGAAGACGGCATACGAGATTCCGTGCGCCGGTCT**CGGCATTCCTGCTGAACCGCTCTTCCGATCT**
PE-PCR-002	CAAGCAGAAGACGGCATACGAGATTGTTTCCCACGGTCT**CGGCATTCCTGCTGAACCGCTCTTCCGATCT**
PE-PCR-003	CAAGCAGAAGACGGCATACGAGATGGTAATGAACGGTCT**CGGCATTCCTGCTGAACCGCTCTTCCGATCT**
PE-PCR-004	CAAGCAGAAGACGGCATACGAGATGAAACTGGGCGGTCT**CGGCATTCCTGCTGAACCGCTCTTCCGATCT**
PE-PCR-005	CAAGCAGAAGACGGCATACGAGATACGGGCTGACGGTCT**CGGCATTCCTGCTGAACCGCTCTTCCGATCT**
PE-PCR-006	CAAGCAGAAGACGGCATACGAGATATGAAGTATCGGTCT**CGGCATTCCTGCTGAACCGCTCTTCCGATCT**
PE-PCR-007	CAAGCAGAAGACGGCATACGAGATACTTATTGTCGGTCT**CGGCATTCCTGCTGAACCGCTCTTCCGATCT**
PE-PCR-008	CAAGCAGAAGACGGCATACGAGATGGCGGGAAACGGTCT**CGGCATTCCTGCTGAACCGCTCTTCCGATCT**
PE-PCR-009	CAAGCAGAAGACGGCATACGAGATACACCTCGGCGGTCT**CGGCATTCCTGCTGAACCGCTCTTCCGATCT**
PE-PCR-010	CAAGCAGAAGACGGCATACGAGATCTCATTGGGCGGTCT**CGGCATTCCTGCTGAACCGCTCTTCCGATCT**

Table S6. 16S rRNA gene taxonomy recovered from the *dsrB*-16S fusion libraries. OTUs were assigned by grouping the 16S rRNA gene sequences into 97% identity clusters. The taxonomy was determined by Qiime based on the Greengenes database. The number of known and novel OTUs and reads recovered is indicated adjacent to taxonomic designations.

Phylogenetic level				*dsrB* OTUs		*dsrB* reads	
Class	Order	Family	Genus	Non-novel	Novel	Non-novel	Novel
Betaproteobacteria	Unclassified	Unclassified	Unclassified	0	1	0	1
Deltaproteobacteria	Unclassified	Unclassified	Unclassified	1	1	3	8
	Desulfarculales	Desulfarculaceae	Unclassified	0	1	0	2
	Desulfobacterales	Desulfobacteraceae	Unclassified	7	6	65689	318139
			Desulfococcus	0	2	0	13
	Desulfuromonadales	Desulfuromonadaceae	Unclassified	1	1	1	3
	Syntrophobacterales	Syntrophaceae	Unclassified	1	0	2	0
			Desulfomonile	9	8	642650	107
		Syntrophobacteraceae	Unclassified	5	3	1000489	1088
			Syntrophobacter	1	1	1	4
Gammaproteobacteria	Alteromonadales	Shewanellaceae	Shewanella	0	1	0	1
	Oceanospirillales	Halomonadaceae	Halomonas	0	6	0	4086

Supplementary References

Giloteaux L, Goñi-Urriza M, Duran R. (2010). Nested PCR and new primers for analysis of sulfate-reducing bacteria in low-cell-biomass environments. *Appl Environ Microbiol* **76**:2856–2865.

Lane DJ. (1991). 16S/23S rRNA sequencing. In:*Nucleic Acid Techniques in Bacterial Systemantics*, Stackebrandt, E & Goodfellow, M (eds), Wiley & Sons: Chinchester, England, pp. 115–175.

Müller AL, Kjeldsen KU, Rattei T, Pester M, Loy A. (2015). Phylogenetic and environmental diversity of DsrAB-type dissimilatory (bi)sulfite reductases. *ISME J* **9**:1152–1165.

Preheim SP, Perrotta AR, Martin-Platero AM, Gupta A, Alm EJ. (2013). Distribution-based clustering: using ecology to refine the operational taxonomic unit. *Appl Environ Microbiol* **79**:6593–6603.

Rodrigue S, Materna AC, Timberlake SC, Blackburn MC, Malmstrom RR, Alm EJ, *et al.* (2010). Unlocking short read sequencing for metagenomics. *PloS One* **5**:e11840.

Schloss PD, Westcott SL, Ryabin T, Hall JR, Hartmann M, Hollister EB, *et al.* (2009). Introducing mothur: open-source, platform-independent, community-supported software for describing and comparing microbial communities. *Appl Environ Microbiol* **75**:7537–7541.

Tamminen MV, Virta MPJ. (2015). Single gene-based distinction of individual microbial genomes from a mixed population of microbial cells. *Front Microbiol* **6**:195.

Vakatov D. (2013). NCBI C toolkit. http://www.ncbi.nlm.nih.gov/IEB/ToolBox/SDKDOCS/INDEX.HTML.

Wagner M, Loy A, Klein M, Lee N, Ramsing NB, Stahl DA, *et al.* (2005). Functional marker genes for identification of sulfate-reducing prokaryotes. *Methods Enzymol* **397**:469–489.

Wagner M, Roger AJ, Flax JL, Brusseau GA, Stahl DA. (1998). Phylogeny of dissimilatory sulfite reductases supports an early origin of sulfate respiration. *J Bacteriol* **180**:2975–2982.

Williams R, Peisajovich SG, Miller OJ, Magdassi S, Tawfik DS, Griffiths AD. (2006). Amplification of complex gene libraries by emulsion PCR. *Nat Methods* **3**:545–550.
