# Supplementary material for: Massively parallel sequencing of single cells by epicPCR links functional genes with phylogenetic markers
Source: ISME J. 2015 Sep 22;10(2):427–36. doi: 10.1038/ismej.2015.124 (PMC4737934; doi:10.1038/ismej.2015.124)
Supplement: Supplementary Figure S2 [file ismej2015124x2.pdf]

Massively parallel sequencing of single cells by epicPCR links functional genes with phylogenetic markers

Supplementary Information README

The supplementary information includes supplementary methods, figures, tables, and references. The index below (also included in the SI file) lists page numbers for each section and subsection. Legends for supplementary figures and tables are included individually after each item.

Supplementary Methods……………………………………………………………… 1
epicPCR Reagents……………………………………………………………….. 1
epicPCR Equipment…………………………………………………………….1
epicPCR Solutions………………………………………………………………1
epicPCR Procedure……………………………………………………………. 2
epicPCR Accessory Procedures………………………………………………4
Sample collection, bulk 16S rRNA gene and *dsrB* gene
library preparation..…………...…………...…………...…………… 6

Supplementary Figures……………………………………………………………….. 9
Figure S1………………………………………………………………………… 9
Figure S2………………………………………………………………………… 10
Figure S3………………………………………………………………………… 11
Figure S4………………………………………………………………………… 12
Figure S5………………………………………………………………………… 13
Figure S6………………………………………………………………………… 14

Supplementary Tables……………………………………………………………… 15
Table S1………………………………………………………………………… 15
Table S2………………………………………………………………………… 16
Table S3………………………………………………………………………… 17
Table S4………………………………………………………………………… 18
Table S5………………………………………………………………………… 19
Table S6………………………………………………………………………… 20

Supplementary References…………………………………………………………... 21
